# A multitask GNN-based interpretable model for discovery of selective JAK inhibitors

**DOI:** 10.1186/s13321-022-00593-9

**Published:** 2022-03-15

**Authors:** Yimeng Wang, Yaxin Gu, Chaofeng Lou, Yuning Gong, Zengrui Wu, Weihua Li, Yun Tang, Guixia Liu

**Affiliations:** Shanghai Key Laboratory of New Drug Design, School of Pharmacy, East China University of Science and Technology, Shanghai, 200237 China

**Keywords:** Selective JAK inhibitors, GNN, Multitask learning, Model interpretations, Key substructures, Applicability domain

## Abstract

The Janus kinase (JAK) family plays a pivotal role in most cytokine-mediated inflammatory and autoimmune responses via JAK/STAT signaling, and administration of JAK inhibitors is a promising therapeutic strategy for several diseases including COVID-19. However, to screen and design selective JAK inhibitors is a daunting task due to the extremely high homology among four JAK isoforms. In this study, we aimed to simultaneously predict pIC_50_ values of compounds for all JAK subtypes by constructing an interpretable GNN multitask regression model. The final model performance was positive, with R^2^ values of 0.96, 0.79 and 0.78 on the training, validation and test sets, respectively. Meanwhile, we calculated and visualized atom weights, followed by the rank sum tests and local mean comparisons to obtain key atoms and substructures that could be fine-tuned to design selective JAK inhibitors. Several successful case studies have demonstrated that our approach is feasible and our model could learn the interactions between proteins and small molecules well, which could provide practitioners with a novel way to discover and design JAK inhibitors with selectivity.

**Graphical Abstract:**

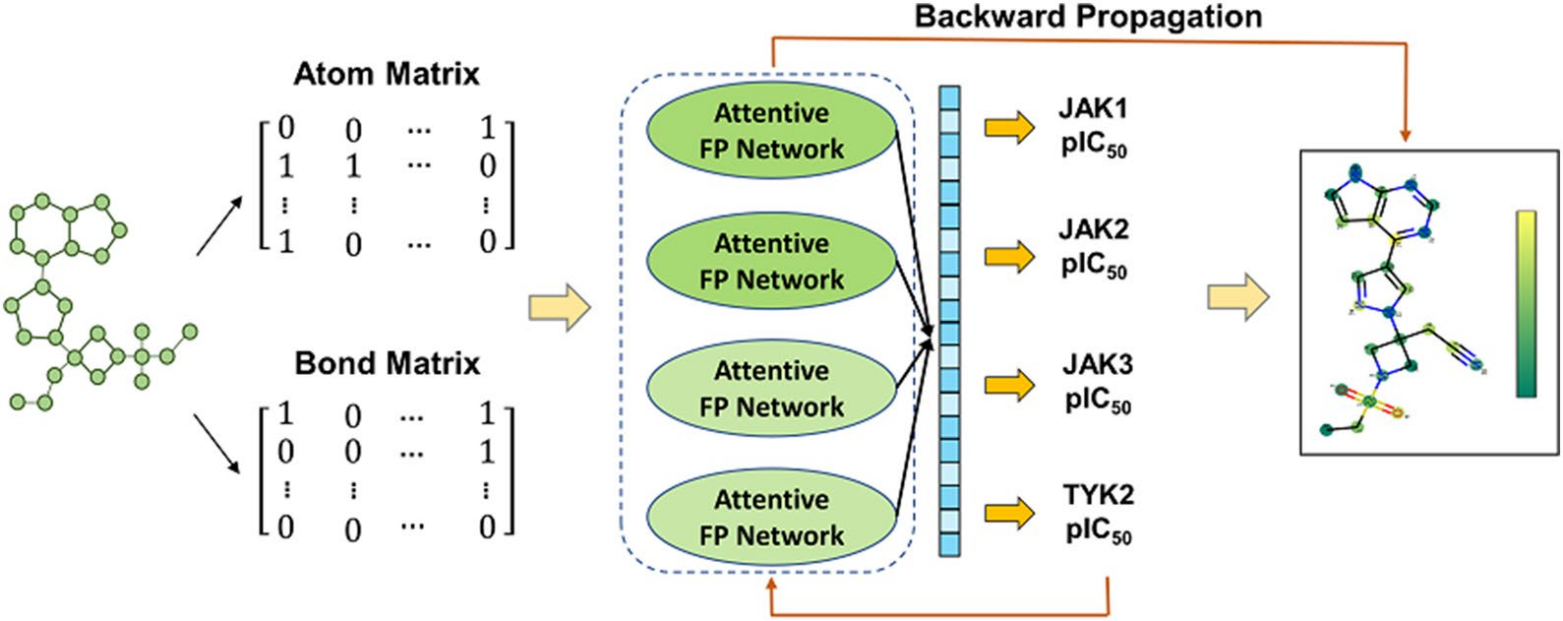

**Supplementary Information:**

The online version contains supplementary material available at 10.1186/s13321-022-00593-9.

## Introduction

Cytokines are small proteins produced and secreted by immune and non-immune cells that are involved in intercellular signaling and interactions [1, 2]. They promote and restrict one another, forming an extremely complex cytokine immune regulatory network [3]. When multiple cytokines released, the balance of the network would be broken, leading to the loss of immune homeostasis and causing a few immune-mediated inflammatory diseases [3]. More seriously, it would result in a cytokine storm that often happened in viral infections like severe acute respiratory syndrome coronavirus 2 (SARS-CoV-2) [4–7]. Cytokine levels could be regulated through the Janus kinase/signal transducers and activators of transcription (JAK/STAT) pathway [8]. When cytokine binds to the receptor outside the cell membrane, the cytokine receptor is activated and phosphorylates the JAK and the downstream molecule STAT. The complex then enters into the nucleus and controls the transcription of cellular genes, which in turn impacts the biological function of the cell [9, 10]. Hence, we could design certain JAK inhibitors for the treatment of immune and inflammatory diseases that were mediated by cytokines.

JAK is a class of non-receptor tyrosine kinases, including four subtypes, namely JAK1, JAK2, JAK3, and TYK2, which regulate different cytokines. Currently, nine JAK inhibitors have been approved by the US Food and Drug Administration (FDA), the European Medical Agency or other regulatory agencies (Additional file 1: Table S1). Nonetheless, a large portion of them are non-selective, having more or less toxicities or other undesirable side effects. For example, tofacitinib is the first pan-JAK inhibitor that targets JAK1, JAK2 and JAK3 for the treatment of moderate or severe active ulcerative colitis (UC), but owing to the simultaneous inhibition of JAK2-mediated erythropoietin and GM-CSF signaling, it has some safety concerns relating to significant adverse reactions including anemia, neutropenia, thrombocytopenia, zoster, and pulmonary embolism [11–13]. The similar story also happened in baricitinib, 10% of patients treated with baricitinib showed side effects such as upper respiratory tract infections and high blood cholesterol levels (hypercholesterolemia) [14]. Therefore, it is reasonable to propose that inhibitors targeting one specific JAK isoenzymes without affecting other JAK-dependent signals could spare toxicities and maximize the clinical benefit. However, discovering small molecules that bind selectively to a given protein target has long been a major stumbling block to modern drug discovery [15]. The promise of in silico screening is tantalizing, as it would allow compounds to be screened at greatly reduced cost [16].

**Fig. 1:**
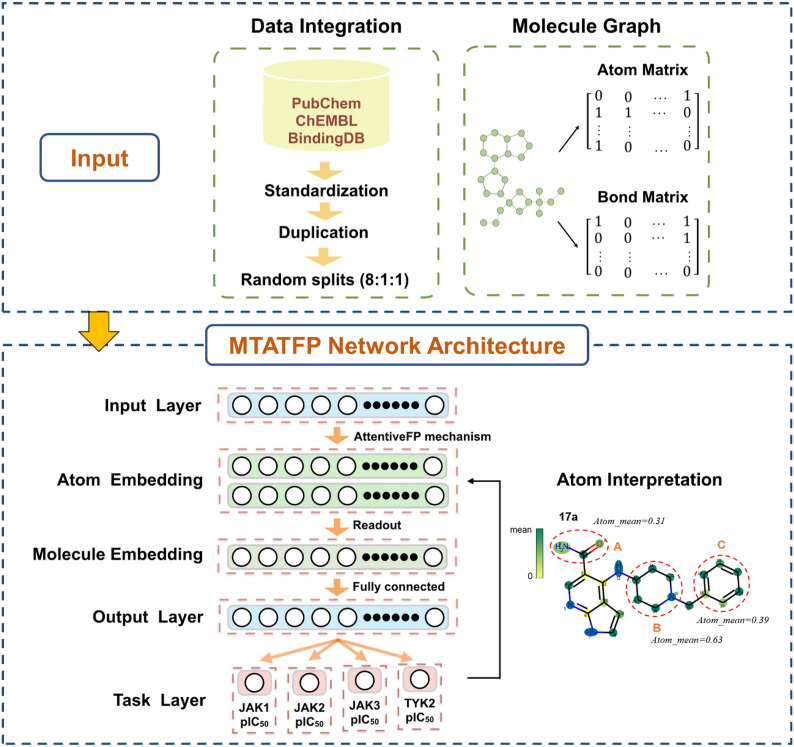
The overview of our work. The main steps include data integration, transformation of molecules into molecular graphs as model input, construction of the MTATFP network and interpretation of molecules by atom weights obtained from back propagation

The common structure of all JAKs consists of four structural domains composed of seven homologous regions [JH1–7] [17]. Most of known small molecular inhibitors targeting JAKs are active site-directed. They bind to the adenosine triphosphate (ATP) site of the catalytic domain (also referred to the JH1 or “Janus Homology 1” domain) [18]. The crystal structures of the JH1 domains have been resolved for the four JAK isoenzymes. However, due to the high sequential homology and structural similarity of the ATP active site across JAK family, it is too hard to discover highly selective molecules for a specific JAK family member by structure-based virtual screening (VS) methods, as Bajusz’s study did [19]. In recent years, deep learning, a branch of machine learning, has been an effective tool for drug discovery, especially in molecular property prediction and ligand-based VS fields [20]. Compared with traditional machine learning methods, deep learning encompasses several layers of stacked complex neural networks that can represent and learn deeper knowledge [21]. Graph neural networks (GNN) have been gaining popularity among many scholars recently. It is plausible for GNN to represent atoms and bonds with nodes and edges, respectively, which are much more powerful at capturing the latent patterns and require less feature engineering efforts [22]. However, deep learning methods required large datasets and are poorly interpretable. To address these issues, we constructed a multitask regression model based on the attentive fingerprint framework (MTATFP) that allows for the simultaneous prediction of IC_50_ values of compounds for the four JAK isoforms (shown in Fig. 1). In addition, we utilized attention coefficients to assign weights to each atom of the compound and visualize them. Our virtual screening platform could facilitate the discovery and modification of JAK inhibitors.

## Methods

### Data collection and processing

In this work, the inhibitors of four JAK isoforms and their experimental IC_50_ values were collected from three databases, PubChem [23], ChEMBL [24] and BindingDB [25] (indicated in Fig. 1). Molecules from different sources were converted to SMILES strings and preprocessed by RDKit [26] and MolVS (https://molvs.readthedocs.io/en/latest/index.html), including normalization of structures, desalting, neutralization of charge and elimination of duplicate molecules (the canonical SMILES strings were chosen as the unique identification and the repeated molecules with lower IC_50_ values were kept). In order to ensure numerical stability during model building, we transformed IC_50_ (nM) into its negative logarithmic scale pIC_50_ (-LgIC_50_).

For developing multitask regression models, the obtained data set was randomly divided into training set, validation set and test set at a ratio of 8:1:1 by python scripts. Training set is used to build the model, validation set is used for hyper-parameter optimization and test set is for the model evaluation. Furthermore, to evaluate the generalization ability of our model, we extracted 33 K_d_ values of JAKs from Davis’s works [27] and 152 activity values from Anastassiadis *et al* [28] (both outside the training set) as two external validation sets. In Anastassiadis’s datasets, we converted the activity values to IC_50_ values with an equation defined earlier [29].

### Multitask learning based on attentive FP network

#### Attentive FP network architecture

As a typical graph convolutional network, graph attention network (GAT) introduced an attention mechanism which has been successfully applied for achieving better neighbor aggregation [30]. The Attentive FP framework was first proposed by Xiong and colleagues [31]. It introduced the graph-attention mechanism to learn both atomic and molecular properties of a given chemical structure, which is tailored for molecular feature extractions. In the present study, we applied the Attentive FP network architecture on four JAK-based tasks to predict the pIC_50_ values (see Fig. 1). We have constructed two Attentive FP convolutional layers for extracting atomic features and a readout layer for molecular embedding, which ultimately output the predicted values using a fully connected layer. A LeakyReLU [32] function was introduced as a nonlinear activation function after a linear transformation.

Before building the attentive FP network, we translated the JAK-related molecules into molecular graphs using RDKit (version 2020_03_1), the rules were shown in Additional file 1: Table S2.

#### Multitask learning strategy

In this work, we employed a multitask learning strategy to improve the predictive performance of small datasets. Four tasks shared the same hidden layers and hyper-parameters during training process, and separated to different tasks at the output of the fully connected layer in the network (shown in Fig. 1). It takes advantage of implicit data augmentation by borrowing similarly structured information features [33]. For example, we could allow model eavesdrop to send JAK2-task to learn some features which were difficult for TYK2-task. Furthermore, it helped to focus the model’s attention on impactful features since other tasks can provide additional evidence whether the features are relevant or not [34]. Meanwhile, we also constructed four single-task models on each JAK subtype for comparison with the multitask model.

#### Model training protocol

Concerning the overwhelming complexity and high computational cost of neural networks, we used random searching strategy based on previous experience for hyper-parameter settings, including a few common ones (such as learning rate, weight decay, batch size) and Attentive FP hyper-parameters (such as the number of network layers, graph feat size and dropout). The performance metrics of validation set were used for model selection. To avoid overfitting and saving computational resources, we used early stopping approaches. A maximum epoch was set as 1000. If the performance metric had not improved in 20 epochs on the validation set, the training process was terminated early. Attentive FP was trained by the Deep Graph Library Python (DGL) package (version 0.6.0) [35] with cuda 10.1 and the DGL-LifeSci [36] extension (github.com/awslabs/dgl-livesci), which ran on the GPU version of the PyTorch [37] framework (version 1.5.0).

#### Atom interpretation

Lack of interpretability is another issue concerned by machine learning and deep learning, usually called “the black-box mode” [38]. Invertion of the Attentive FP model by extracting the hidden layers or attention weights could provide access to the model’s interpretation, which would help chemists gain insights into the skyrocketing volume and complexity of drug discovery data. In the Attentive FP network, since atoms are treated as nodes in the molecular graph, we could obtain atom weights by gradient backward propagation and visualized according to Fig. 1 (the darker the color of the node is, the greater the impact on the target is). Based on this, we could identify atomic features that are significant for the target and then optimize chemical structures for drug design. Taking the multitask model into account, the atoms having relatively high contributions play big roles for all tasks. Here, we believe that these high scoring atoms are crucial for the selectivity of the JAK family. The process of atom weighting calculation could be formulated below:

1 $$Atom_{m,n} = \frac{{{\text{exp}}\left( {LeakyReLU\left( {\left. {\vec{a}^{T} \left[ {W\vec{h}_{k} ||W\vec{h}_{j} } \right]} \right)} \right.} \right.}}{{\mathop \sum \nolimits_{{i \in N_{i} }} {\text{exp}}\left( {LeakyReLU\left( {\left. {\vec{a}^{T} \left[ {W\vec{h}_{k} ||W\vec{h}_{i} } \right]} \right)} \right.} \right.}}$$

This formula expresses the contributions of node *j* (the neighbor node, a neighbor atom) for node *k* (the target node, a specific atom) without considering the graph structural information. ${h}_{k}$ is the state vector of node *k*, ${h}_{j}$ is the state vector of node *j* and $W$ is a trainable weight matrix. The mutual attention mechanism $\overrightarrow{a}$ ($W{\overrightarrow{h}}_{k}$,$W{\overrightarrow{h}}_{j}$) is applied in the model, parameterized by the weight vector $\overrightarrow{a}$ and activated by applying LeakyReLU. The transpose is denoted as *T* and concatenation is by ||. The numerator is expressed as the sum of the weights of all neighbor nodes of *k*.

To determine whether built models have learned the protein binding logic, 38 molecular series were extracted and compiled from Park’s works [39]. These molecular series were experimentally synthesized JAK inhibitors and contained activity values for all JAK isoforms. We performed predictions with a trained model for these inhibitors, inspecting and visualizing the atomic weights for fine-tuning analysis. Our goal was to evaluate how faithfully these contributions captured the binding logic for each JAK proteins.

### Machine learning approaches

To further enable methodology evaluation, we compared our multitask deep learning model with a state-of-the-art machine learning method called LightGBM [40], which is a distributed gradient boosting framework based on the decision tree algorithm. LightGBM is faster and lower memory consumption in contrast to Extreme Gradient Boosting (XGBoost) [41] that is another gradient boosting framework. It abandons the Level-wise decision tree growth strategy and used a Leaf-wise strategy with a depth limit. Level-wise is an inefficient algorithm because it treats the leaves of the same level indiscriminately, which brings a few unnecessary computational overheads since many leaves indeed have low splitting gain and there is no need to search and split. By contrast, Leaf-wise is more productive, which finds the leaf with the highest splitting gain and then splits it, and so on. Moreover, LightGBM adds a maximum depth limit on top of Leaf-wise to prevent overfitting while maintaining high efficiency. Given the above considerations, we applied LightGBM algorithm rather than XGBoost. The ECFP4 fingerprint (1024 bits) [42] was chosen for the input of the machine learning model and calculated by PaDEL-descriptor program (version 2.13) [43], which is widely used in QSAR/QSPR tasks to characterize molecules. Furthermore, considering that better performance may be achieved by other types of molecular representations [44], we calculated the molecular descriptors of the compounds using RDKit as a second approach. The model parameters we set to build eight regression models were also based on random search, and the final outcomes were illustrated in Additional file 1: Table S3. In order to proceed model performance comparisons, we guaranteed the consistency of the all datasets (training set, validation set and test set) with deep learning methods.

### Model evaluation

The measurements in these data sets are quantitative. We built regression models for the quantitatively measured data sets. The performance of regression models was evaluated by the following metrics: R^2^ (the degree of concordance between the predictions and the corresponding observations), MAE (mean absolute error) and RMSE (root-mean-square error). The predictive model could be single-task or multitask. For the multitask models, we calculated the performance metrics for each individual task and reported their average values as global metrics. The formulas are as follows.

2 $$R^{2} = 1 - \frac{{\mathop \sum \nolimits_{i} \left( {\widehat{{y_{i} }} - y_{i} } \right)^{2} }}{{\mathop \sum \nolimits_{i} \left( {\overline{{y_{i} }} - y_{i} } \right)^{2} }}$$

3 $$MAE = \frac{1}{m}\mathop \sum \limits_{i = 1}^{m} \left| {\left( {y_{i} - \widehat{{y_{i} }}} \right)} \right|$$

4 $$RMSE = \sqrt {\frac{1}{m}\mathop \sum \limits_{i = 1}^{m} \left( {y_{i} - \widehat{{y_{i} }}} \right)^{2} }$$

where, $y_{i}$ presented true values, $\widehat{{y_{i} }}$ presented predicted values and *m* is the total number of data sets.

### Y-randomization testing

To estimate the impacts of chance correlation, y-randomization was used, which is developed to validate a given regression model and initially proposed by Rücker et al. [45]. In this approach, activity values of four tasks are randomly shuffled, to disrupt the relationship between label values and feature values in the training data, and construct the model on the basis of unordered data. The procedure is repeated number of times. Ideally, if the new regression models have lower R^2^ values for several trials, then the given model is thought to be robust. Inversely, given a regression model, even though the performance of the model is great respect to the training data, if the performance after y-randomization is good as well, then there should be chance correlations in the data set and the model could be overfitted. In the present study, we randomly shuffled the IC_50_ values of training sets and validation sets ten times based on different random seeds. These disordered data were then applied to reconstruct the attentive FP multitask model repetitively, and recorded the R^2^ values of the training and validation set for each y-randomization model.

### Definition of applicability domain

Defining the applicability domain (AD) of the model is a key component in the five standards of OECD (Organization for Economic Co-operation and Development) on QSAR models, which can be considered as the chemical space of the modeling compound data [46]. Thus, we adopted a methodology based on structural similarity named the Euclidean distance-based method (DM) for AD analysis in this study. The chemical structures were represented by Morgan fingerprints. This method will ultimately obtain a distance threshold (${D}_{T}$) which can determine whether the compound is within the AD of the model. The detailed formula is as follows:

5 $$D_{T} = d_{ave} + Z*\theta$$

where, $d_{ave}$ is the average of the Euclidean distance between each compound and its nearest compound in the training set, $\theta$ is the corresponding standard deviation, $Z$ is an optional parameter representing the significance level. First, we calculate the structural similarity between the test set and the training set of compounds by RDKit to get $d_{ave}$ and $\theta$, then keep the k nearest neighbor molecules with the highest similarity as the distance value. If one of these k distances exceeds the threshold of $D_{T}$, the compound is considered to be outside of domain (OD) [47]; otherwise, it has fallen into the domain (ID).

## Results and discussion

### Data analysis

**Fig. 2:**
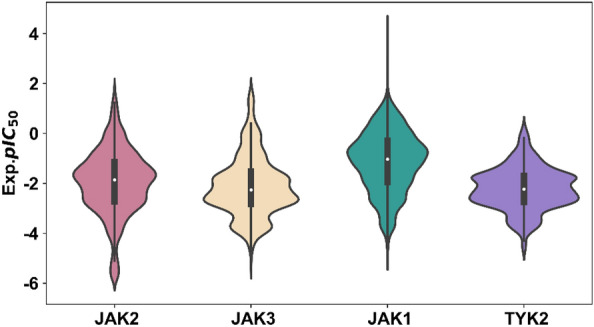
The box-whiskers plots concerning experimental pIC_50_ values of four JAK datasets (JAK1, JAK2, JAK3 and TYK2)

After data processing, 13,898 compounds (including 8087 JAK1, 10,828 JAK2, 4485 JAK3 and 2465 TYK2 inhibitors) were retained to build multitask regression models. Most of the compounds have more than two JAK isoform experimental IC_50_ values ranging from 0.00125 nM to 767,000 nM. The final experimental value distributions of each JAK isoform dataset were shown in Fig. 2, which was adequate to build a robust activity prediction model.

### Chemical diversity analysis

**Fig. 3:**
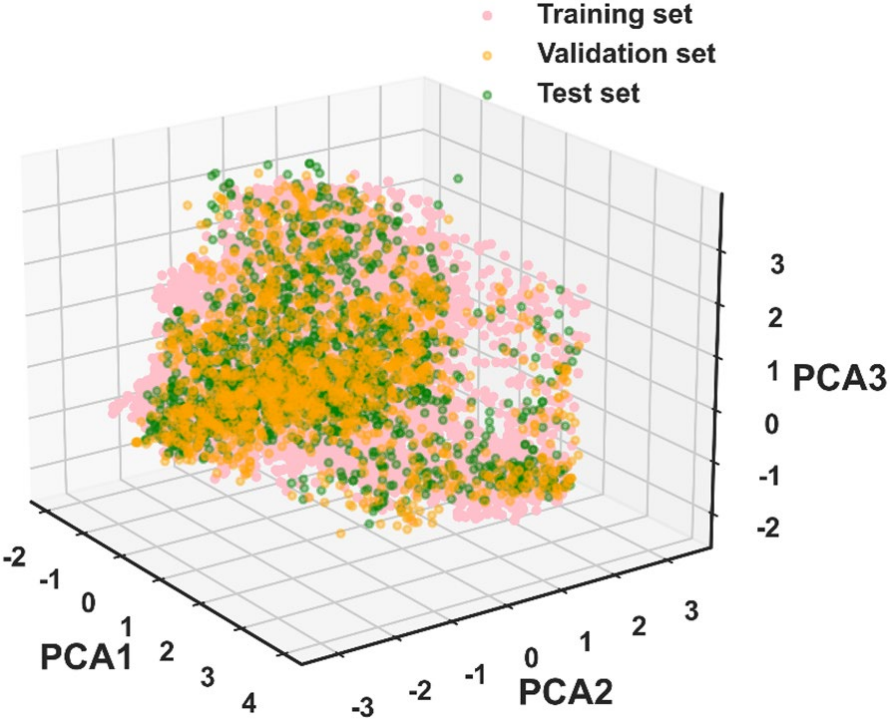
Three-dimensional spatial scatter plots of the chemical spatial distributions on the MACCS fingerprint features for the training set, validation set and test set, represented as the first three principal components of the PCA of the small molecular JAK inhibitors

To verify the diversity of collected compounds and rationalization of data set partitioning, we visualized the chemical space with a principal component analysis (PCA) [48]. MACCSkeys generated by RDKit as input of PCA were used to represent JAK ligands. As demonstrated by the chemical space defined by the first three principal components in Fig. 3, a wide distribution of scatters was observed, indicating a high diversity of collected molecules. Besides this, the chemical space of the validation and test sets were completely overlapped with the chemical space of the training set, which implied justifications of data splits. In our model, the training set contained 10,824 molecules, the validation set contained 1529 molecules, and there were 1545 molecules in the test set.

### Task relevance analysis

**Fig. 4:**
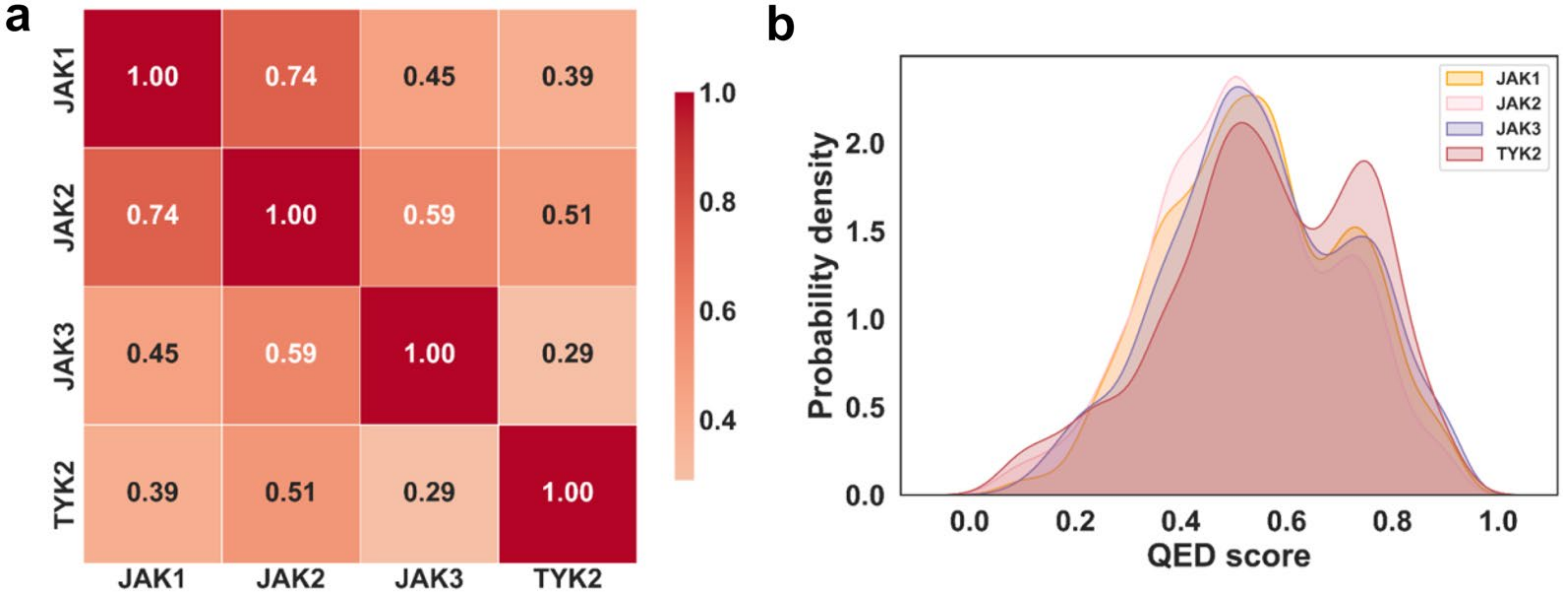
Relevance analysis on individual endpoint values and input features for the full data. **a** Heat map showing the correlation among the pIC_50_ values of the four JAK subtype small molecular inhibitors, darker colors indicated higher relevance. **b** The QED fraction distributions of the small molecules in the four tasks are plotted, the more overlapped regions the more compatible the compounds are

In contrast to transfer learning, multitask learning is more suitable for data sets that have shared molecules with related tasks and can predict different tasks using only one network architecture [49]. To verify that there are some correlations between the individual tasks, we conducted a correlation analysis on the pIC_50_ values of the four tasks, and the findings were shown in Fig. 4a. We also calculated the quantitative estimate of drug-likeness (QED) values for the compounds in the four tasks, and plotted their area under curves in Fig. 4b to estimate the similarity of the compounds, with larger overlap areas reflecting higher compound similarity across the four tasks. QED value is a quantitative metric for assessment of drug-likeness ranging from 0 (all properties unfavorable) to 1 (all properties favorable) [50]. It could be expected that the activity values of all four tasks were moderately correlated and the four compound datasets were resembled as well.

### Optimization of MTATFP model

The prediction performance of our multitask attentive FP (MTATFP) model on the validation dataset was summarized in Additional file 1: Figure S1. In this case, since the size of our overall dataset was relatively small compared to other tasks used for deep learning, and GNN was prone to overfitting [51], we only set two Attentive FP layers and one pooling layer here, and the number of neurons was set to the default value of 300. In the light of the model's parameter search results, we noticed that batch size and dropout had a relatively slight effect on the model, while the learning rate had a significant effect. Models with lower values of learning rate always produce weak predictive power. A large learning rate might cause big prediction fluctuations without learning enough knowledge, while a model with a small learning rate might demand excessive updates to achieve convergence. The best set of hyper-parameters for each category of tasks obtained from the previous random searching process was used to train the most predictive model. Ultimately, we trained models for 217 epochs (according to early stopping state strategy illustrated in Additional file 1: Figure S2) with a batch size of 256 samples, and employed the Adam [52] optimizer with a learning rate of 1e-3 and a weight decay of 1e-6. Parameters in the network were updated using MSELoss which measure mean-squared error as loss functions of the regression tasks. The top performing model had an R^2^ value over 0.8 and the model was selected for the next step in the test set evaluation.

### Model performance

**Fig. 5:**
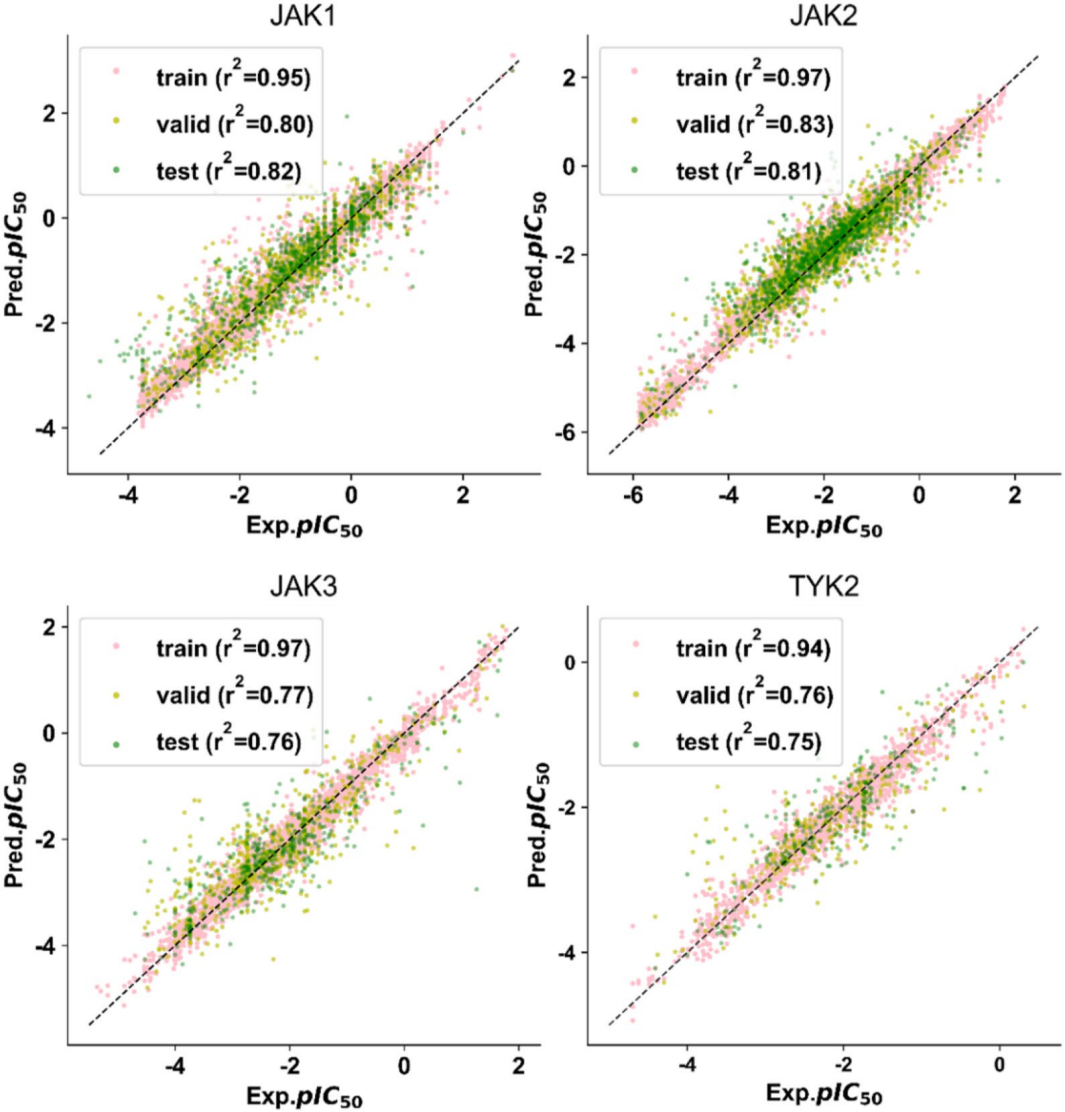
Linear scatter plot of the training, validation and test set in our MTATFP model. The closer the scatter points are to the straight line, the better the approximation of the model predictions to the true values is

**Fig. 6:**
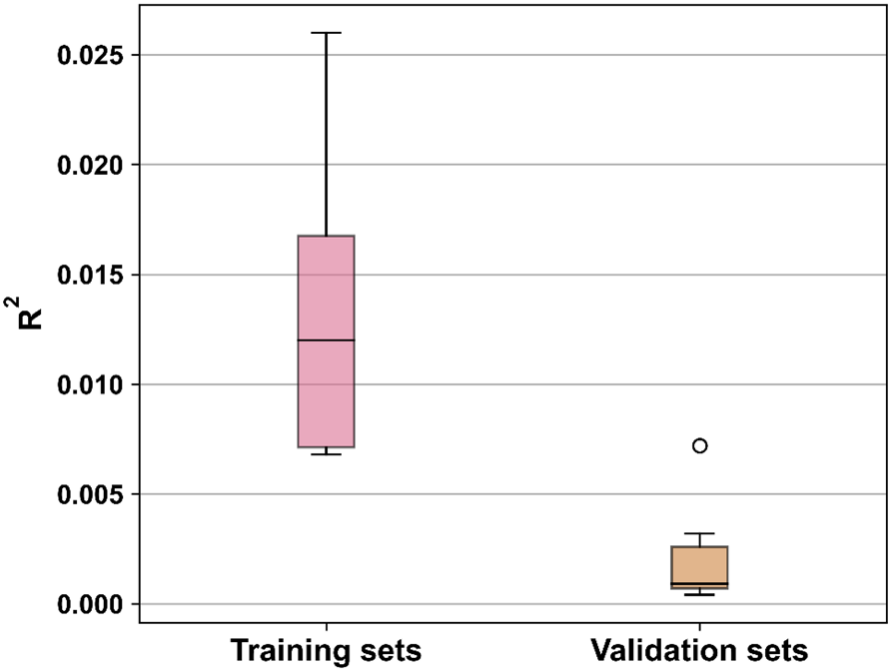
Box plots of the results of ten times Y randomization tests on the training and test sets. The closer the R^2^ value was to 1, the better the model performed

MTATFP models showed predictive capabilities according to Fig. 5 and Table 1, with the global R^2^ values of 0.96, 0.79 and 0.78 calculated on the training, validation and test sets, respectively. Additionally, the global MAE values of those three datasets (training, validation and test sets) are 0.15, 0.37 and 0.37 (Table 2). The corresponding global RMSE values are 0.23, 0.51 and 0.52 (Table 3). Distributed to every individual task in our model, the R^2^ values in training set from 0.20 to 0.27. And in validation set and test set the R^2^ values ranged from 0.75 to 0.82, the MAE values ranged from 0.30 to 0.42 and the RMSE values ranged from 0.42 to 0.58. These values indicated that the difficulty of the training task varies. The MTATFP regression model was further validated by the Y-randomization test (Fig. 6), and the global R^2^ values of the randomized model was apparently lower than those of the non-randomized model, which demonstrated that the given regression model is robust and not the outcome of chance. Although none of the tasks showed perfect predictive power and the performance on small datasets were worse in some extends, the results are remarkably better than random (proved by Y-Randomization Test), indicating that meaningful molecular graph features related to target endpoints were identified during the learning process.

**Table 1:** R^2^ performance of each model on the four tasks

	Methods	Training set	Validation set	Test set
Global	MTATFP	0.96	0.79	0.78
	STATFP	0.93	0.75	0.73
	LightGBM_MD	0.93	0.69	0.70
	LightGBM_ECFP4	0.91	0.71	0.70
JAK1	MTATFP	0.95	0.80	0.82
	STATFP	0.95	0.80	0.80
	LightGBM_MD	0.91	0.75	0.72
	LightGBM_ECFP4	0.91	0.75	0.76
JAK2	MTATFP	0.97	0.83	0.81
	STATFP	0.96	0.82	0.8
	LightGBM_MD	0.94	0.70	0.75
	LightGBM_ECFP4	0.92	0.75	0.71
	Xgboost^a^	0.97^a^	0.80^a^	0.80^a^
JAK3	MTATFP	0.97	0.77	0.76
	STATFP	0.91	0.68	0.70
	LightGBM_MD	0.92	0.69	0.70
	LightGBM_ECFP4	0.90	0.69	0.73
TYK2	MTATFP	0.94	0.76	0.75
	STATFP	0.91	0.69	0.63
	LightGBM_MD	0.93	0.61	0.62
	LightGBM_ECFP4	0.91	0.65	0.61

It showed that the performance of various models (deep learning methods based on MTATFP or STATFP strategies and LightGBM-based machine learning approaches) on each task. The closer the R^2^ value was to 1, the better the model performed

^a^is the best results in Yang’s work. Although each dataset could not be guaranteed to be the identical, our multitasking model has obvious advantages as well

**Table 2:** MAE performance of each model on the four tasks

	Methods	Training set	Validation set	Test set
Global	MTATFP	0.15	0.37	0.37
	STATFP	0.21	0.40	0.40
	LightGBM_MD	0.22	0.45	0.44
	LightGBM_ECFP4	0.23	0.43	0.43
JAK1	MTATFP	0.19	0.37	0.39
	STATFP	0.18	0.36	0.39
	LightGBM_MD	0.25	0.45	0.44
	LightGBM_ECFP4	0.24	0.42	0.44
JAK2	MTATFP	0.18	0.38	0.38
	STATFP	0.21	0.40	0.40
	LightGBM_MD	0.22	0.48	0.47
	LightGBM_ECFP4	0.25	0.46	0.46
JAK3	MTATFP	0.16	0.42	0.41
	STATFP	0.27	0.49	0.47
	LightGBM_MD	0.24	0.49	0.48
	LightGBM_ECFP4	0.26	0.48	0.45
TYK2	MTATFP	0.15	0.30	0.30
	STATFP	0.19	0.34	0.35
	LightGBM_MD	0.15	0.36	0.38
	LightGBM_ECFP4	0.18	0.37	0.37

The lower the MAE value was, the better the model performed

**Table 3:** RMSE performance of each model on the four tasks

	Methods	Training set	Validation set	Test set
Global	MTATFP	0.23	0.51	0.52
	STATFP	0.29	0.56	0.57
	LightGBM_MD	0.30	0.62	0.61
	LightGBM_ECFP4	0.33	0.60	0.60
JAK1	MTATFP	0.27	0.52	0.53
	STATFP	0.27	0.51	0.55
	LightGBM_MD	0.34	0.61	0.60
	LightGBM_ECFP4	0.35	0.57	0.60
JAK2	MTATFP	0.26	0.51	0.54
	STATFP	0.28	0.55	0.56
	LightGBM_MD	0.31	0.66	0.62
	LightGBM_ECFP4	0.36	0.63	0.64
JAK3	MTATFP	0.21	0.58	0.58
	STATFP	0.36	0.70	0.66
	LightGBM_MD	0.33	0.67	0.65
	LightGBM_ECFP4	0.38	0.66	0.62
TYK2	MTATFP	0.20	0.44	0.42
	STATFP	0.26	0.49	0.52
	LightGBM_MD	0.21	0.52	0.55
	LightGBM_ECFP4	0.25	0.52	0.52

The lower the RMSE value was, the better the model performed

The tables (Tables 1, 2 and 3) also displayed the assessment results of all single-task models based on Attentive FP (STATFP) networks or LightGBM algorithm. Obviously, studies on four homologous proteins’ IC_50_ predictions have shown that multitask learning had great advantages over single-task learning and outperformed other methods on both validation and test set. In the case of STATFP models that utilized the same framework, the global R^2^ for the validation and test set of the MTATFP model improved by 5% and 7%, the global RMSE decreased by 10% and 8%, and the global MAE decreased by 8% and 8%, respectively. Observing the performance of individual tasks in the multitask model, it had significantly improved the model performance on the small data of JAK3 and TYK2, while keeping the model validity constant or even slightly enhancing on the large data set of JAK1 and JAK2 (probably learned the molecular graph knowledge from other tasks), which was in accordance with our anticipations of employing the multitask learning strategy. Versus deep learning methods, the LightGBM method based on molecular descriptors or fingerprints seemed to have close predictive abilities on the small data of JAK3 and TYK2, yet the similar results were not achieved in larger data set. All in all, the MTATFP model yielded the best performance in all validation and test evaluations, producing the most accurate predictions with better generalizability.

### Comparison with other studies on JAK selectivity

**Fig. 7:**
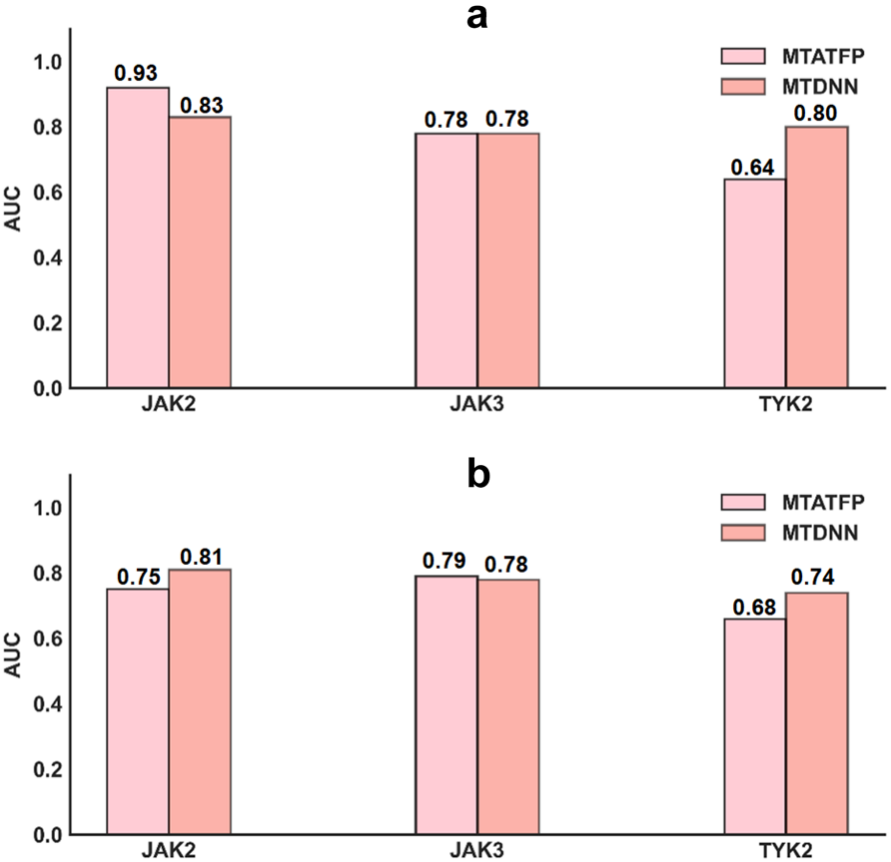
Histograms of AUC comparison between MTATFP model and MTDNN model. **a** The model performance in Davis’s datasets including JAK2, JAK3 and TYK2. **b** The model performance in Anastassiadis’s datasets. The closer the AUC value was to 1, the better the model performed

Recently, Li and colleagues proposed a multitask classification model of 391 kinases including JAK2, JAK3 and TYK2 to distinguish inhibitors from non-inhibitors and achieved good performance [53]. But the classification models did not present accurate bioactivity data and were somewhat insufficient to judge whether a compound was selective or not. For instance, some compounds are selective, where their IC_50_ values for individual kinase isoforms are all below the threshold of 1 μM, but in fact they have a difference multiplicity greater than twice. Or, molecules determined to be selective by the classification model, some of which have IC_50_ values around the threshold, are indeed non-selective compounds. To facilitate the comparison with Li's work, we maintained the evaluation metrics and divided the predicted IC_50_ values into active and inactive compounds at 1 μM to assess their AUC values. For Davis’s datasets, a K_d_ values over 1 μM was defined as signifying inactivity. All thresholds were set in consistent with the literature and the results of the comparisons were represented in Fig. 7. It could be seen that our MTATFP model had similar performance compared to Li's MTDNN model in the discriminations of inhibitors for the respective JAK members. Besides, we could extraordinarily provide specific bioactivity values IC_50_ on top of that. Although our model performed slightly worse on several data sets, this was likely associated with the chemical space of the data set with certain compounds occurring outside the AD of the model (shown in Additional file 1: Figure S3). Overall, MTATFP still exhibited impressive performance on these external datasets, and it would be possible to further improve the generalizability of the model by expanding the size of the training dataset and increasing the diversity of compounds.

In addition, there have been a few other ligand-based VS approaches like other machine learning algorithms and three-dimensional quantitative structure–activity relationship (3D-QSAR) analyses used in the discovery of JAK inhibitors as well, however they only consider drug-likeness but not drug-selectivity since these efforts only gave modeling analysis of IC_50_ values for a specific JAK isomer. Yang and colleagues constructed three groups of regression models based on fingerprints and XGBoost methods to acquire highly potent JAK2 kinase inhibitors [54], the results were shown in Table 1. In contrast to their work, our model exhibited identical predictive capability for JAK2 active small molecules and at the parallel it was able to present the IC_50_ values of compounds for the other three isoforms with selectivity analysis. The most important thing is that our model breaks the black box by visualizing the atomic weights for model interpretations rather than just giving some prediction outputs. And compared with several 3D-QSAR works for JAK selectivity studies [55–58] which collected only tens or hundreds of compounds to model, our model has a wider AD and higher robustness because of the broader chemical space of the four datasets.

### Atom visualization and interpretation

**Fig. 8:**
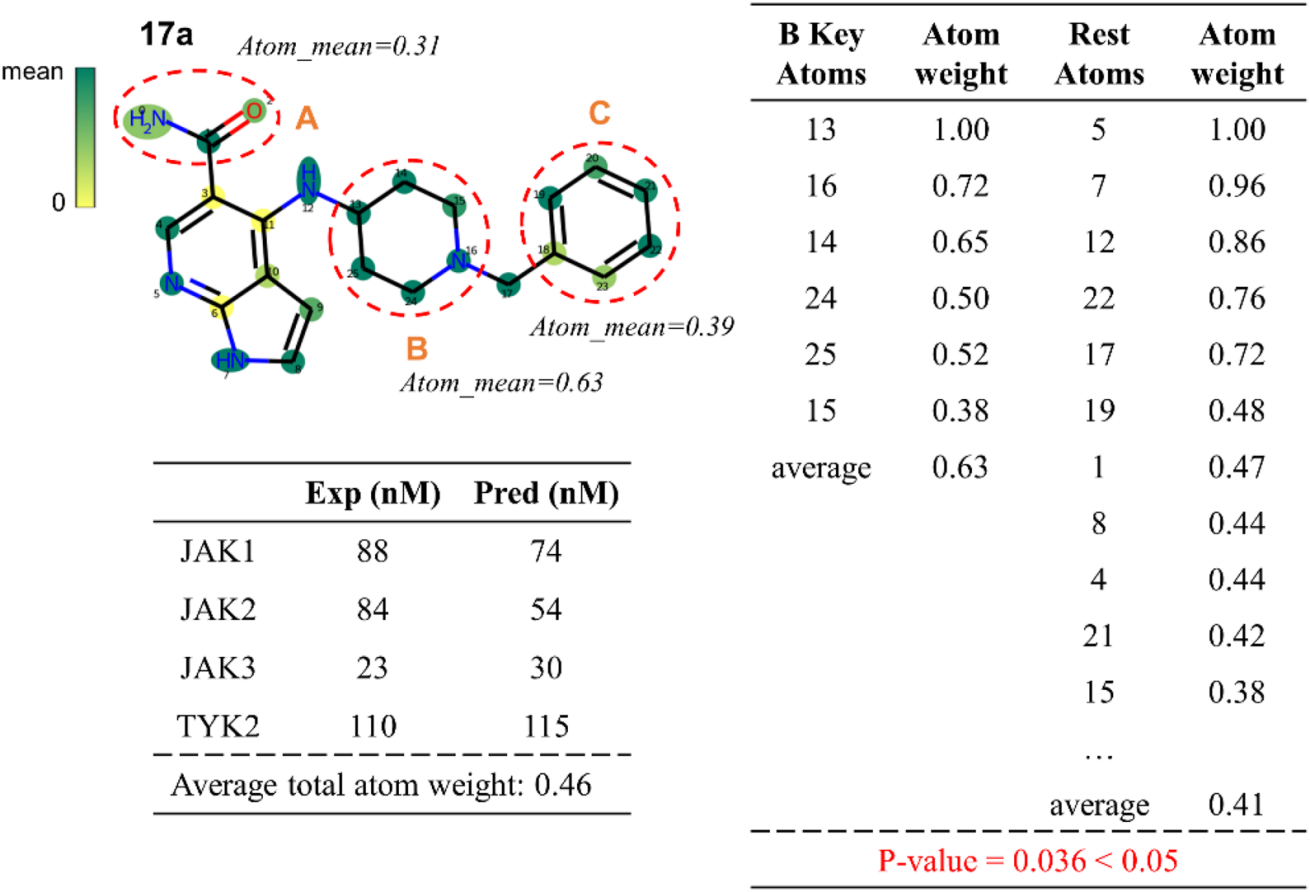
The atom weight visualization of the lead compound **17a**. Three areas (A, B, C) were chosen to be modified for the selective JAK1 inhibitor design. Atom_mean represented the average of the atom weights in the highlighted region in red and the deeper the color in the graph is, the higher the value of the atom weights is. The table in the figure exhibited some of the atom weight values with their atom indexes and the predicted IC_50_ values

In this part of the study, we defined atoms with weight score above the average as key atoms, and molecular fragments with multiple key atoms were considered as key substructures. Altering a few key atoms or substructures might have a meaningful impact on the predicted outcomes of four tasks. To estimate whether the atom weightings were statistically significant, we introduced a rank sum test [59]. Two sets of atom weightings were tabulated as A and B. A was the atom weightings in the substructure that we deemed important, and B was the weightings of the remaining atoms in that molecule. In a rank sum test, if the hypothesis that A is much greater than B is valid, the p-value is less than 0.05 which means the atoms in A are much more important than B. To facilitate the presentation of those findings, the atom weightings of each molecule in JAK inhibitor series were regularized and visualized. Three chosen areas were modified to heighten the selectivity of JAK1 inhibitors in Park’s works (shown in Fig. 8), some of which would be interpreted by our trained multitask models. We predicted the IC_50_ values of the lead compound **17a** for the four JAK isoforms and calculated its atom weights. As shown in Fig. 8, the cyclohexane in the B part showed a dark green color, which we assumed to be a key substructure, and performed a rank sum test on the atoms in it and found that the hypothesis held and the average atomic weight (0.63) of this substructure was also remarkably higher than that (0.41) of the remaining atoms, so we determined that the cyclohexyl amine is a key substructure. Although the average atom weights of the A and C parts was a bit lower than the total atom weight (0.46), they were also potential to affect the final outcomes due to the presence of some key atoms, which was consistent with the descriptions in the literature and we could utilize these for drug design.

**Fig. 9:**
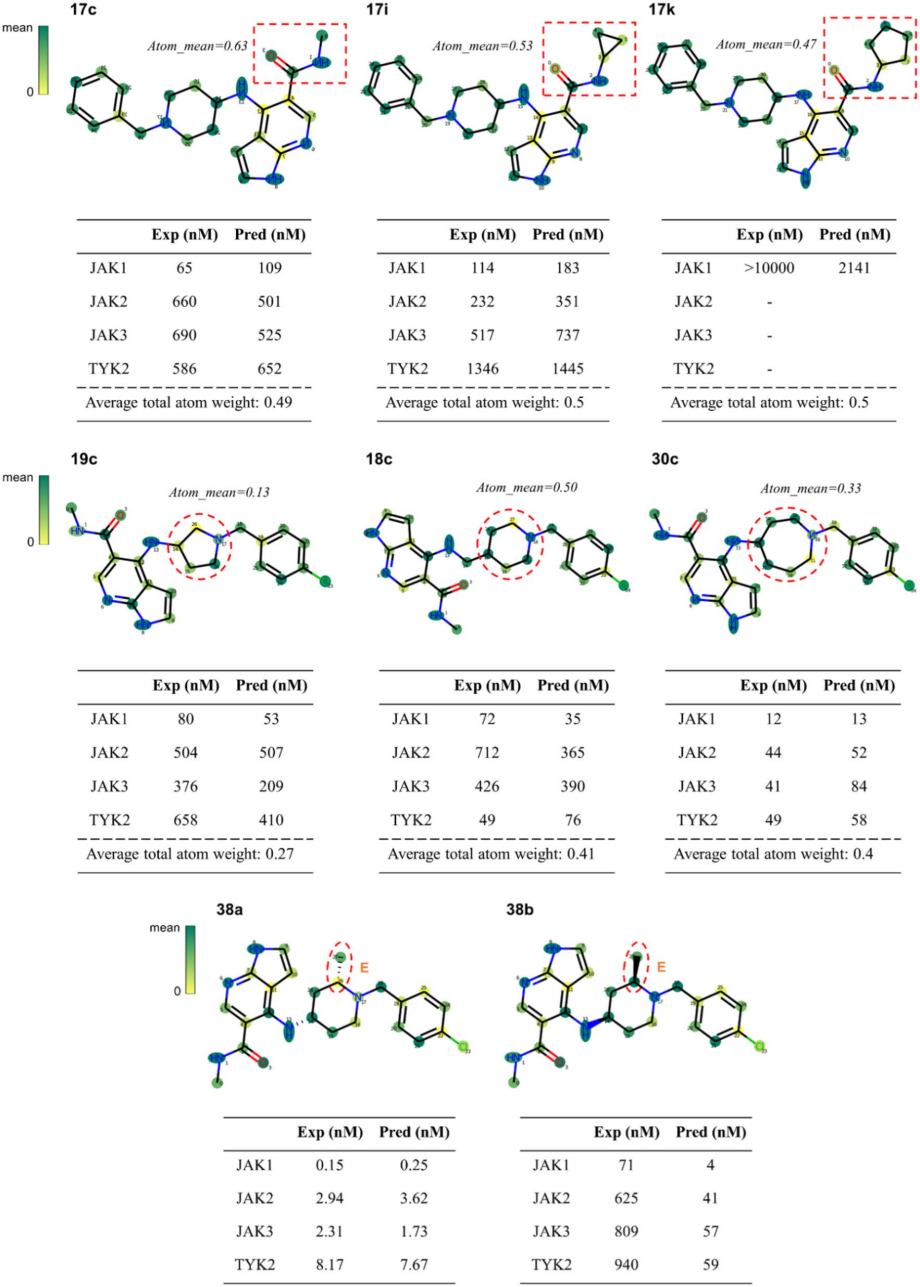
Several examples of atom weight visualization for JAK inhibitor small molecules

The JAK1 selective inhibitors were designed by fine-tuning the substituents or scaffolds and observing the deviation of the four IC_50_ values. Methyl amide, cyclopropyl amide and cyclopentyl amides were compared in the A part, and it was found that the amide bonds of all three compounds (**17c**, **17i** and **17 k,** shown in Fig. 9) were displayed in dark color. However, the average atom weights of A part decreased as the amide volume became bulkier and the binding affinity of the compounds to JAK changed at the same time, suggesting that amide might be a JAK isomer selective switch especially the methyl amide due to it had the highest atom weight and the greatest influence. Our model predictions were in agreement with experimental results in the literature [39]. We further investigated the effect of the size and shape of the cyclic amine group in the B part on the selectivity of JAK by determining three basic scaffolds and simultaneously ensuring that the other substituents were the same, including 3-aminopyrrolidine (**19c**), piperidin-4-ylmethyl (**18c**) and 4-aminoazepane (**30c**) (presented in Fig. 9). The results showed that the inhibitory activity against JAK1 was slightly increased but selectivity decreased obviously with the size of the cyclic amine group, which also supported that the cyclic amine group was a key substructure in concordance with the literature descriptions [39]. At next stage, we were interested in investigating whether our model could correctly capture the stereochemistry of the compounds and whether the cis–trans isomers of the compounds were correlated with the selectivity of the JAK inhibitors. According to the literature [39], **38a** was more selective for JAK1 than JAK2, JAK3 and TYK2, respectively, while the enantiomer **38b** has a higher IC_50_ value for JAK1 which was far outweighed by **38a**. Under our calculations, although our model had some variations in the predictions for **38b**, the overall tendency was the consistency with the experimental results, and the weights of the atoms near one of the **E** bonds were changed to some extent, which indicated that our model have learned certain knowledge of chemical space.

### Analysis of applicability domain

The AD setting can avoid over-prediction bias arising from the significant characteristic differences between test and training chemicals, which means that it is necessary to evaluate the AD of a model to identify the reliability of the prediction for different molecules [60]. By calculating the distance matrix, we obtained ${d}_{ave}$ of 0.1262 and $\theta$ of 0.2522. Next, in order to find the best AD, we set different k values and $Z$ values, finally received the corresponding threshold ${D}_{T}$ and the corresponding number of OD compounds, which were given in Table 4. As observed, the consequent increase in $Z$ values and decrease in k resulted in an accompanying growth of ${D}_{T}$ and a constant decline in the compounds outside the AD. Subsequently, we used our MTATFP model to predict the ID and OD compounds in the test set at different values of k and ${D}_{T}$, and the performance of each data set was shown in Table 5. By comparing and analyzing the results, we noticed that the overall evaluation metrics of the model were all improved when ${D}_{T}$ = 0.06315 (k = 3, $Z$=− 0.25) (compared to the complete test set without removing the OD compounds) and were able to distinguish to the maximum extent between ID and OD compounds (the prediction performance of ID compounds was significantly better than that of OD compounds). The findings indicated that our defined AD is appropriate for the proposed MTATFP model and could allow the model to serve more accurately in practical applications.

**Table 4:** The amounts of compounds outside the applicability domain in the test set at different $Z$ and *k* values

$Z$	− 0.25	− 0.2	− 0.15	− 0.1
k				
3	130	92	67	43
4	176	117	87	51
5	226	150	114	69
${D}_{T}$	0.06315	0.07576	0.08837	0.1010

**Table 5:** The evaluation performance of compounds both inside domain (ID) and outside the domain (OD) in the test set at different $Z$ and *k* values

	*R*^2^		MAE		RMSE	
	ID	OD	ID	OD	ID	OD
k3 $Z$\0.25	0.79	0.72	0.36	0.48	0.51	0.63
k4 $Z$\0.25	0.79	0.76	0.36	0.44	0.51	0.59
k5 $Z$\0.25	0.79	0.74	0.36	0.44	0.50	0.59
k3 $Z$\0.2	0.79	0.73	0.37	0.45	0.51	0.59
k4 $Z$\0.2	0.79	0.74	0.36	0.45	0.51	0.59
k5 $Z$\0.2	0.79	0.75	0.36	0.44	0.51	0.59
k3 $Z$\0.15	0.79	0.70	0.37	0.45	0.51	0.57
k4 $Z$\0.15	0.78	0.76	0.37	0.45	0.51	0.58
k5 $Z$\0.15	0.79	0.75	0.36	0.44	0.51	0.58
k3 $Z$\0.1	0.78	0.74	0.37	0.44	0.52	0.57
k4 $Z$\0.1	0.78	0.77	0.37	0.44	0.52	0.57
k5 $Z$\0.1	0.79	0.75	0.37	0.45	0.51	0.59

In addition, it is worth to notice that our MTATFP model not only has an excellent prediction ability for ID compounds (R^2^ = 0.79, MAE = 0.36, RMSE = 0.51), but also has a good prediction performance for OD compounds (R^2^ = 0.72, MAE = 0.48, RMSE = 0.63). We believed that this phenomenon could reflect the advantage of a multitask learning strategy, which allows subtasks to learn from each other. Even if a compound is missing information from one task,  it will still be determined by stealing knowledge from other tasks, which may correspondingly increase the fault tolerance of the model.

## Conclusions

Designing selective JAK inhibitors has been a daunting challenge owing to the extremely high homology among individual isoforms. Although traditional QSAR models tend to have perfect predictive power, there is still no guarantee that the predictions could guide selective drug design. Here, we constructed an MTATFP regression model to predict the pIC_50_ values of small molecules to four JAK isoforms and got atom weights to identify key atoms and substructures important to the target selectivity. Then we used these key substructures to fine-tune the compounds, and further defined the applicability domain of the model. The results indicated that the constructed model could effectively learn the interactions between small molecule ligands, each JAK isoform and the key substructures recognized can correctly guide JAK-selective inhibitor design. The multitask learning strategy also significantly improved the model performance for small data sets, giving an extended error tolerance. Overall, our JAK-selective virtual screening platform offers the advantages of speed, accuracy and interpretability for quantitative prediction of selective JAK inhibitors.

## Supplementary Information

**Additional file 1: Table S1**. Approved JAK inhibitors and their current indications. **Table S2**. Some molecular graph features computed with RDKit. **Table S3**. Parameters’ settings of LightGBM based model for four tasks. **Figure S1**. Heat map of optimal hyper-parameters search for a MTATFP model. Here, the default parameters were learning rate (0.1, 0.01, 0001 and 0.0001), drop out (0.2, 0.3, 0.4, 0.5), and search for parameters at different batch size (64, 128, 256) to determine the best ones. The darker the color is, the better the R^2^ value of the validation set is. **Figure S2**. A curve graphs of Loss and R^2^ during training process on multitask models. Early stopping criterion for training is that the R^2^ on validation set is no longer improving in 20 epochs and get the best epoch 217 eventually. **Figure S3**. The chemical spatial distributions of the training, Davis and Anastassiadis dataset. It represented as the first three principal components of the PCA of the JAK small molecular inhibitors.

## Data Availability

The software used to execute the study is freely available as follows: PaDEL-Descriptor (http://padel.nus.edu.sg/software/padeldescriptor). Attentive FP framework was trained with the Deep Graph Library Python (DGL) package (version 0.6.0) [35] with cuda 10.1 and the dgllife extension (github.com/awslabs/dgl-livesci), which ran on the GPU version of the PyTorch framework (version 1.5.0) [37]. Molecular structures were handled and translated into molecular graphs using RDKit (version 2020_03_1) [26]. All the codes, trained models, and datasets are available on our GitHub (https://github.com/Yimeng-Wang/JAK-MTATFP).

## Abbreviations

SARS-CoV-2: Severe acute respiratory syndrome coronavirus 2
JAK/STAT: Janus kinase/signal transducers and activators of transcription
FDA: Food and drug administration
UC: Ulcerative colitis
ATP: Adenosine triphosphate
VS: Virtual screening
QSAR: Quantitative structure–activity relationship
GNN: Graph neural networks
MTATFP: Multitask attentive FP
GAT: Graph attention network
XGBoost: Extreme gradient boosting
MAE: Mean absolute error
RMSE: Root-mean-square error
AD: Applicability domain
DM: Distance-based method
OD: Outside of domain
ID: Inside of domain
PCA: Principal component analysis
QED: Quantitative estimate of drug-likeness
STATFP: Single-task models based on Attentive FP
