# Supplementary material for: A multitask GNN-based interpretable model for discovery of selective JAK inhibitors
Source: J Cheminform. 2022 Mar 15;14:16. doi: 10.1186/s13321-022-00593-9 (PMC8922399; doi:10.1186/s13321-022-00593-9)
Supplement: Supplementary file 1 — Additional file 1: Table S1. Approved JAK inhibitors and their current indications. Table S2. Some molecular graph features computed with RDKit. Table S3. Parameters’ settings of LightGBM based model for four tasks. Figure S1. Heat map of optimal hyper-parameters search for a MTATFP model. Here, the default parameters were learning rate (0.1, 0.01, 0001 and 0.0001), drop out (0.2, 0.3, 0.4, 0.5), and search for parameters at different batch size (64, 128, 256) to determine the best ones. The darker the color is, the better the R2 value of the validation set is. Figure S2. A curve graphs of Loss and R2 during training process on multitask models. Early stopping criterion for training is that the R2 on validation set is no longer improving in 20 epochs and get the best epoch 217 eventually. Figure S3. The chemical spatial distributions of the training, Davis and Anastassiadis dataset. It represented as the first three principal components of the PCA of the JAK small molecular inhibitors. [file 13321_2022_593_MOESM1_ESM.pdf]

SUPPLEMENTARY MATERIAL

A Multitask GNN-based Interpretable Model for Discovery of Selective JAK Inhibitors

Yimeng Wang, Yaxin Gu, Chaofeng Lou, Yuning Gong, Zengrui Wu, Weihua Li, Yun Tang*, Guixia Liu*

Shanghai Frontiers Science Center of Optogenetic Techniques for Cell Metabolism, Shanghai Key Laboratory of New Drug Design, School of Pharmacy, East China University of Science and Technology, Shanghai 200237, China

* To whom correspondence should be addressed.

Tel: +86-21-64250811

Fax: +86-21-64251033

Email: gxliu@ecust.edu.cn (G. Liu); ytang234@ecust.edu.cn (Y. Tang)

Table S1. Approved JAK inhibitors and their current indications

Drug	Target	Indication	Structures
**Baricitinib**	JAK1/JAK2	RA (EMA approved), COVID-19 (EUA)	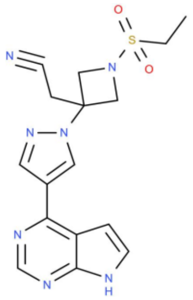
**Ruxolitinib**	JAK1/JAK2	MPN, acute GVHD RA (Japan approved)	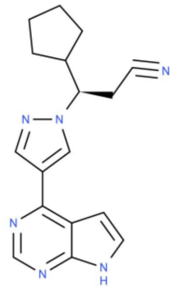
**Oclacitinib**	JAK1	Allergic dermatitis (FDA approved)	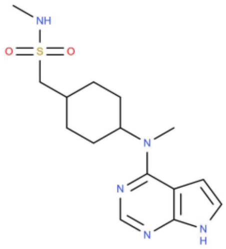
**Tofacitinib**	JAK1/JAK2/JAK3	RA (FDA approved, EMA approval recommended), PsA, UC	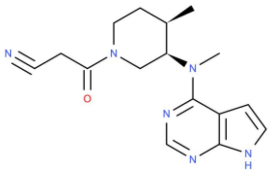
**Upadacitinib**	JAK1	RA (FDA approved), PsA (approved in EMA), AS (approved in EMA)	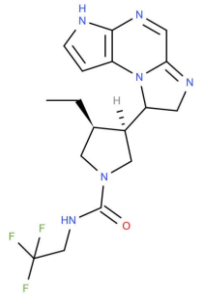
**Delgocitinib**	Pan-JAK	AD (approved in Japan)	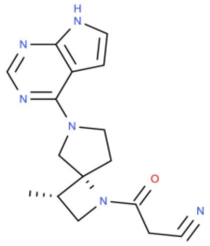

**Filgotinib**	JAK1	RA (approved in EU, Japan)	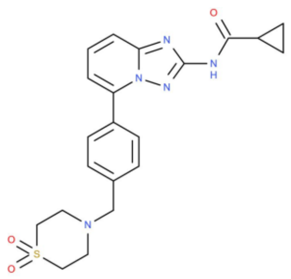

AD, atopic dermatitis; EUA, Emergency Use Authorization; GVHD, graft-versus-host disease; MPN, myeloproliferative neoplasms; PsA, psoriatic arthritis; RA, rheumatoid arthritis; UC, ulcerative colitis.

Table S2. Some molecular graph features computed with RDKit[1, 2]

Atom Feature	Size	Description
atom symbol	16	[B, C, N, O, F, Si, P, S, Cl, As, Se, Br, Te, I, At, metal] (one-hot)
degree	6	number of covalent bonds [0,1,2,3,4,5] (one-hot)
formal charge	1	electrical charge (integer)
radical electrons	1	number of radical electrons (integer)
hybridization	6	[sp, sp2, sp3, sp3d, sp3d2, other] (one-hot)
aromaticity	1	whether the atom is part of an aromatic system [0/1] (one-hot)
hydrogens	5	umber of connected hydrogens [0,1,2,3,4] (one-hot)
chirality	1	whether the atom is chiral center [0/1] (one-hot)
chirality type	2	[R, S] (one-hot)
**Bond Feature**	**Size**	**Description**
bond type	4	[single, double, triple, aromatic] (one-hot)
conjugation	1	whether the bond is conjugated [0/1] (one-hot)
ring	1	whether the bond is in ring [0/1] (one-hot)
stereo	4	[StereoNone, StereoAny, StereoZ, StereoE] (one-hot)

Table S3. Parameters' settings of LightGBM based model for four tasks

	**Learning rate**	**N estimators**	**Num leaves**	**Max depth**	**Min child samples**
JAK1_MD	0.2	173	19	7	5
JAK2_MD	0.4	295	15	4	8
JAK3_MD	0.3	101	16	5	14
TYK2_MD	0.2	92	19	6	8
JAK1_ECFP4	0.2	376	18	5	10
JAK2_ECFP4	0.2	405	18	5	9
JAK3_ECFP4	0.5	95	12	4	7
TYK2_ECFP4	0.2	165	15	4	6

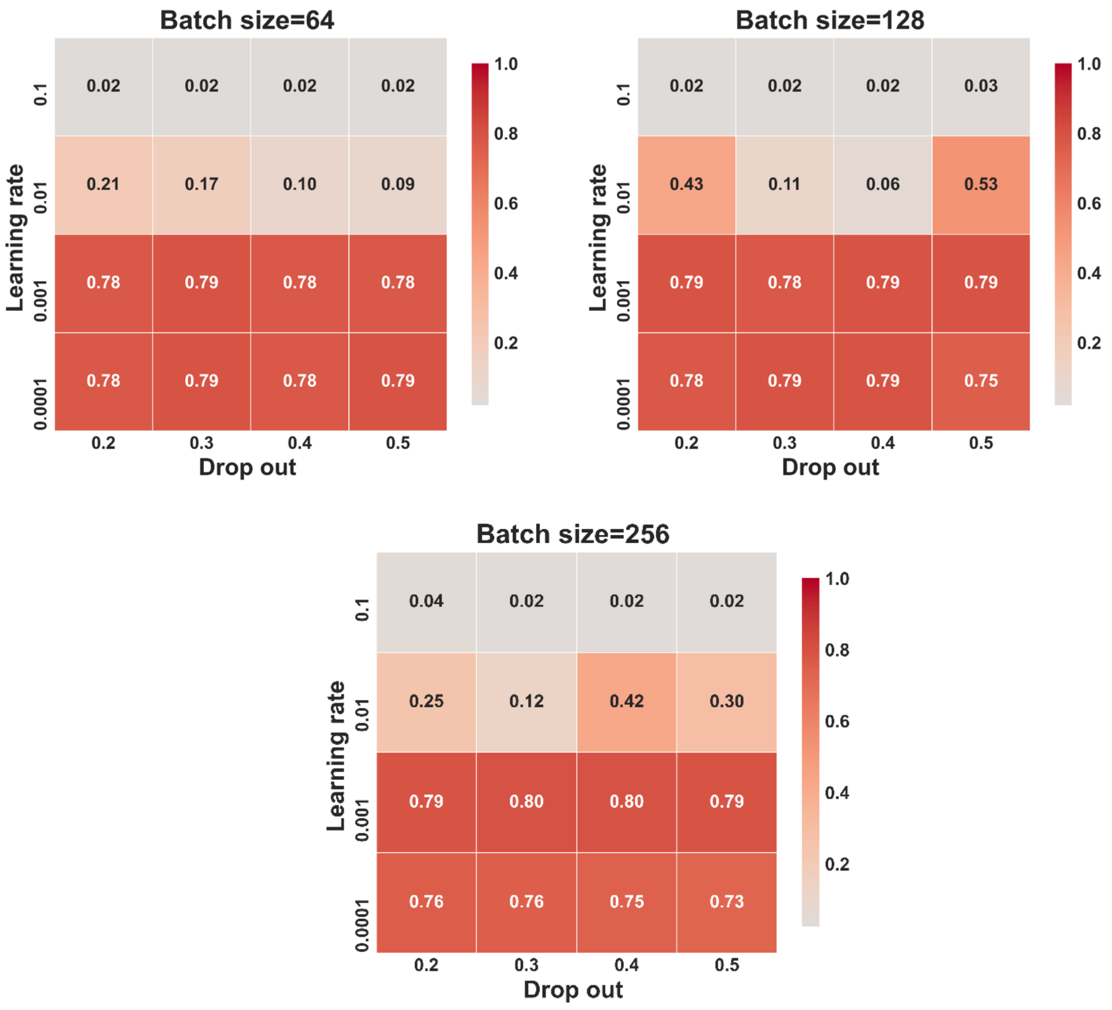

Figure S1. Heat map of optimal hyper-parameters search for a MTATFP model. Here, the default parameters were learning rate (0.1, 0.01, 0001 and 0.0001), drop out (0.2, 0.3, 0.4, 0.5), and search for parameters at different batch size (64, 128, 256) to determine the best ones. The darker the color is, the better the R^2 value of the validation set is.

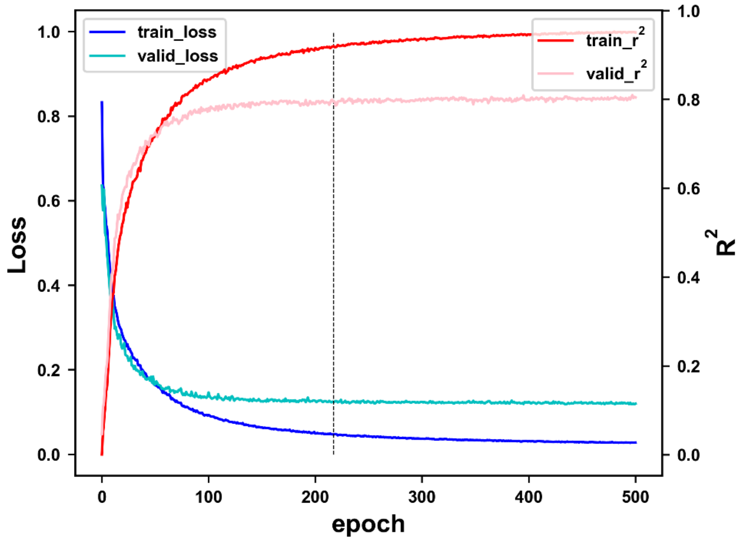

Figure S2. A curve graphs of Loss and R^2 during training process on multitask models. Early stopping criterion for training is that the R^2 on validation set is no longer improving in 20 epochs and get the best epoch 217 eventually.

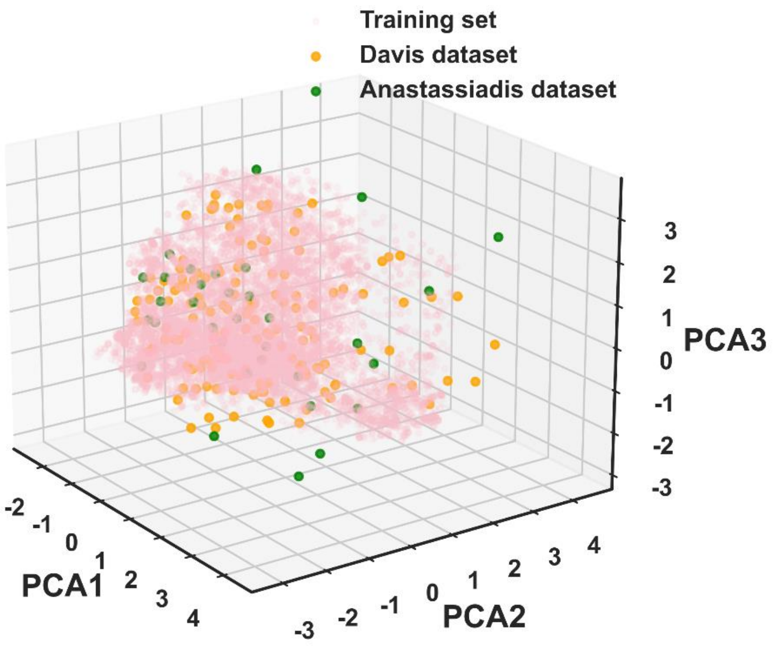

Figure S3. The chemical spatial distributions of the training, Davis and Anastassiadis dataset. It represented as the first three principal components of the PCA of the JAK small molecular inhibitors.

References

1. Landrum G, Tosco P, Kelley B (2020) rdkit/rdkit: 2020_03_1 (Q1 2020) Release 10

2. Xiong Z, Wang D, Liu X et al (2020) Pushing the boundaries of molecular representation for drug discovery with the graph attention mechanism. J Med Chem 63(16):8749-8760
